# Knowledge sharing among academics from Egyptian medical schools during the COVID-19 pandemic

**DOI:** 10.1186/s12909-024-05502-2

**Published:** 2024-06-01

**Authors:** Amany Mohamed Elsayed, Emad Isa Saleh, Mohamed Mahmoud El-Kassas, Haidy Samir Khalil

**Affiliations:** 1 Information Science Department, Faculty of Arts, Helwan University, Helwan, Egypt; 2 Endemic Medicine Department, Faculty of Medicine, Helwan University, Helwan, Egypt; 3 Medical Microbiology & Immunology Department, Faculty of Medicine, Helwan University, Helwan, Egypt

**Keywords:** Knowledge sharing, COVID-19, Egypt, Medical sciences, Medical knowledge, Healthcare knowledge, Academic staff, Medical schools.

## Abstract

**Background:**

Sharing knowledge among scientists during global health emergencies is a critical issue. So, this study investigates knowledge-sharing behavior and attitude among staff members of 19 Medical schools in Egyptian universities during the COVID-19 pandemic.

**Methods:**

Across-sectional study was conducted using a web-based questionnaire. A total of 386 replies from the 10,318 distributed questionnaires were analyzed. Descriptive statistics were computed using SPSS (version 22) to summarize the demographic data. Inferential statistics such as the independent and chi-square test were used to achieve the study aims.

**Results:**

More than half of the respondents (54.4%) indicated that their levels of knowledge of COVID-19 were good. Most participants (72.5%) reported that scientific publications and international websites were the most reliable source of their knowledge concerning COVID-19. More than 46% stated they sometimes share their knowledge. The lack of time to share and organizational culture were the most important factors that could affect their knowledge sharing. Additionally, about 75% of participants shared knowledge about treatment.

**Supplementary Information:**

The online version contains supplementary material available at 10.1186/s12909-024-05502-2.

## Introduction

Some pandemics and epidemics have been related to health diseases for several decades. The COVID-19 pandemic is an emerging virus that has brought about many critical issues facing all medical and healthcare-related institutions and workers. As WHO [1] reported on Jul 15, 2022, there have been 557,917,904 confirmed cases of COVID-19, including 6,358,899 deaths. In July 2022 the WHO Director-General said the COVID-19 pandemic was nowhere near over [2]. The unknown nature of the virus and the lack of sufficient experience made knowledge sharing an essential part of fighting against COVID-19 and the global efforts to control it. Physicians share knowledge in a multi-layered way, as they are in contact with patients, managers, nurses, and each other. Abijah [3] states that the only source of optimism considering the current lack of a COVID-19 treatment is just knowledge sharing, which focuses on prevention.

Collecting and sharing scientific data during global health emergencies is not a new phenomenon, but efforts are being made to share explicit knowledge of the type of data and research results among scientists, such as the Zika open data initiative. As indicated by Sambo [4], during a pandemic outbreak of the Ebola virus in West Africa, it was crucial to research to fill the knowledge gap about the natural history of the virus, therapy, and prevention. In 2008 the global initiative to share all influenza data was established; now, it is recognized as a reliable and effective platform for exchanging published and unpublished influenza data [5]. In 2020, the WHO Research and Development Blueprint [6] created the go-to platform for scientific collaboration among scientists, developers, regulators, and funders across the globe to hasten crucial pandemic research, including examinations, vaccinations, treatments, and more. In addition, it has been arranged for the world’s best scientific minds to analyze data before publication. These emerging efforts mean that sharing knowledge is an effective way to respond to COVID-19.

### Medical science and knowledge sharing

In medical science, the doubling period of knowledge was predicted to be 50 years in 1950, 7 years in 1980, and 3.5 years in 2010. It is expected to be 0.2 years—just 73 days—in 2020 [7]. Academic communities like universities and research institutes were the primary source of knowledge. Different types of knowledge are created in universities through education, research, discussion, and clinical learning. Universities play a vital role in creating and transferring scientific knowledge, and Medical science is an affluent area of knowledge. Well-timed use of up-to-date medical knowledge can improve patient and system outcomes [8]. Medical science is one of many practical fields for sharing knowledge about diseases, diagnoses, and therapy, especially among professionals and practitioners. Various techniques and tools for sharing are being implemented, but success depends on the individual’s willingness to transfer knowledge and the prevailing culture in the institution.

In addition, Medicine is a high-risk field in which a mistake or failure can have significant implications, especially in periods of uncertainty. As a result, the most current knowledge must be implemented in this domain. Faculty members in the faculties of Medicine must be research oriented; some were required to work at the COVID-19 frontline. Most of them have been affected by COVID-19 not only because of fear of getting infected or being an infectious carrier but also because of worries about their ability to carry out their academic and research responsibilities. Medical science researchers in each country strive to share information about their research results through international publications. Knowledge sharing is a form of academic communication activity conducted by scholars regarding different COVID-19 treatments [9]. In recent times, countries all over the world have been focusing on healthcare and unstudied diseases. Many studies assume that it is the most knowledge-intensive industry in the world. So, various types of necessary knowledge are supposed to be shared during the ongoing COVID-19 pandemic, such as clinical practices, preventive measures, diagnosis, and treatment.

The employees’ knowledge sharing behavior in any profession is affected by their attitude towards sharing knowledge, subjective norms, and perceived behavioral control. According to Abidi [10], in everyday practice, the sharing of healthcare knowledge takes place in problem-solving conversations among healthcare colleagues. They share knowledge about a particular clinical case, evaluate general interest in a study, clinical status, clinical guideline, or administrative policy, grouping formulas of a guideline or workflow, referral to an expert on the subject, published evidence, assessment, and information to treat or maintain a patient’s health.

Many studies are being done to investigate knowledge, attitudes, and practices among health professionals concerning factors that affect their attitudes and behaviors toward sharing knowledge. One of the first serious discussions conducted by Ryu [11] concluded that subjective norms and attitudes were found to have the most potent total effects on behavioral intentions to physicians’ knowledge-sharing behavior in tertiary hospitals in Korea. Another study by Adem [12] showed a lack of formal and informal knowledge-sharing opportunities at Felege Hiwot Referal Hospital in Ethiopia. According to Abdul Rahman [13], researchers and officers within the National Institutes of Health in Malaysia have a poor culture of knowledge-sharing practice.

COVID-19 astounded the whole globe, including the Arab world. A study by Shaikhain et al. [14] revealed that most healthcare workers in Saudi Arabia followed COVID-19 news from official authorities including the SMOH and the WHO. A recent study [15] revealed that Lebanese physicians have good knowledge about COVID-19. The primary source of their information was the World Health Organization (WHO), followed by the Ministry of Public Health. Al Demour et al. [16] found that physicians in Jordan and Palestine had adequate knowledge of COVID-19; also, WHO websites and scientific journals are trustworthy scientific information sources. A study by Chereka et al. [17] revealed that more than half of healthcare providers at specialized teaching hospitals in Northwest Ethiopia had a good level of COVID-19 knowledge-sharing practice. There is a relatively small body of Saudi literature in the Arabic language concerned with knowledge management application requirements in Jeddah’s Hospitals [18] and the role of the community of practice in supporting health knowledge management [19]. In addition, the role of organizational culture in activating the sharing of tacit knowledge on the faculties of Medicine at King Abdulaziz University in Jeddah [20].

Since the COVID-19 pandemic began, medical education has been severely disrupted [21]. So, this study is the first to date in the Arab world to give significant insights into knowledge-sharing behavior among staff members of faculties of Medicine in Egyptian universities during the COVID-19 pandemic. This study is important because it will illustrate how knowledge is shared and the factors affecting this process. Results from the study will shed light on several aspects of knowledge sharing among academic staff during pandemics. Also, the findings would contribute to the local and global actions taken to control the COVID-19 pandemic and similar future threats. The primary research objective sought to explore the knowledge sharing behaviors of staff members in faculties of Medicine in Egyptian universities during the COVID-19 pandemic. The three sub-objectives sought to gain further insight into the primary research objective by exploring the types of knowledge and challenges they encountered when sharing their knowledge.

The following research objectives guide this study:

1. To examine the knowledge sharing behavior of faculty members in the Egyptian faculties of Medicine during the COVID-19 pandemic.
2. To discover the nature of knowledge shared among faculty members in the Egyptian faculties of Medicine during the COVID-19 pandemic.
3. To identify critical challenges faculty members face when sharing knowledge in the Egyptian faculties of Medicine during the COVID-19 pandemic.

## Methods

A cross-sectional survey was performed in 19 Medical schools in Egyptian universities using an anonymous online questionnaire. The study involved a web-based questionnaire created using Google Forms. A total of 10,318 faculty members of the faculties of Medicine in Egyptian universities (who have email addresses on their academic web pages) were invited to complete the questionnaire. It consists of two parts: Part one creates a demographic profile of the participants, including six questions about the university, gender, age, job title, department, and experience. Part two contains thirteen questions concerning knowledge-sharing practices. All questions were closed-ended questions. The questionnaire items were based on a focus group with the research team, and literature review, customized to meet our study objectives [11, 22, 33, 38].

Both are two kinds of scientific departments: The basic sciences in the study of Medicine, including physiology, anatomy, pathology, microbiology, biochemistry, pharmacology, histology, and clinical departments, which study several medical specialties in the form of a course dedicated to general surgery, internal Medicine, pediatrics, obstetrics and gynecology, orthopedics, emergency Medicine, otolaryngology, ophthalmology, and neurosurgery.

Potential participants were faculty members of medical schools in Egyptian governmental universities. Some are in contact with COVID-19 patients, and others are not. Those who contact COVID-19 patients can be divided into two groups: (1) those who manage cases, chest, endemic Medicine, radiology, internal Medicine, and ICU departments. (2) Those who may see COVID-19 patients during their routine daily work, obstetrics, general surgery, urology …. etc.

### Ethical approval

was obtained before conducting the study from the Research Ethics Committee for Human and Animal Research at the Faculty of Medicine, Helwan University (No. 17-2021). Then, Participants were emailed via the principal investigator’s academic email, asking them to complete the online questionnaire. Survey data was collected over the period from April to December 2021. The consent form was on the first page of the questionnaire to explain what would be asked of participants and if they agreed or disagreed to participate in the research. The respondent will have to click “Yes” to begin the survey. If the respondent clicks “No,” the survey goes immediately to the exit page. Table 1 highlights survey respondents by university.

**Table 1:** Survey respondents by university

University	No. of emails	Responses	Percentage
Faculty of Medicine, Ain Shams University	68	17	4.4
Faculty of Medicine, Al-Azhar University (Boys & Girls)	71	12	3.1
Faculty of Medicine, Alexandria University	39	8	2.1
Faculty of Medicine, Assiut University	2762	2	0.5
Faculty of Medicine, Aswan University	303	7	1.8
Faculty of Medicine, Benha University	2440	21	5.4
Faculty of Medicine, Beni Suef University	698	19	4.9
Faculty of Medicine, Fayoum University	524	25	6.5
Faculty of Medicine, Helwan University	157	65	16.8
Faculty of Medicine, Kafrelsheikh University	131	4	1
Kasr Al-Ainy Faculty of Medicine, Cairo University	145	54	14
Faculty of Medicine, Mansoura University	305	21	5.5
Faculty of Medicine, Menoufia University	24	7	1.8
Faculty of Medicine, Minia University	893	19	4.9
Faculty of Medicine, Sohag University	1149	28	7.3
Faculty of Medicine, South Valley University	276	6	1.6
Faculty of Medicine, Suez Canal University	28	3	0.8
Faculty of Medicine, Tanta University	282	54	14
Faculty of Medicine, Zagazig University	23	14	3.6
**Total**	**10,318**	**386**	**100.0**

It is important to note here that there are 23 governmental faculty of Medicine in Egypt [23], and four were excluded from the survey. Port Said and Armed Forces College of Medicine because they presented only staff names and academic degrees. Moreover, Suez University’s faculty of Medicine has no website. The Faculty of Medicine at Damietta University is the newest college established, and study begins during the academic year 2021/2022, so its website was incomplete.

To increase the response rate, follow-up reminders via email were sent to participants after the initial contact. We received 386 replies from the 10,318 distributed questionnaires; and their answers are analyzed in this study. The response rate was 5%, which is considered low. Many studies assume that actors affect survey response rates, and surveys of Medicine and healthcare workers (academic physicians and nonacademic) have low participation rates. Poor participation is a common problem in such areas, and specialty influences participation rates [24]. The interests of participants may influence the response rate, and the authors think the survey topic may be a niche for them. Another reason is that using commercial email addresses instead of institutional ones is reported as a critical issue in the survey. According to Shannon and Bradshaw [25], internet surveys have disadvantages, such as poor response rates.

It is worth noting that five universities (Suez Canal, Menoufia, Ain Shams, Al-Azhar (Boys & Girls), and Alexandria) have few emails for some department heads and faculty. Cairo and Zagazig did not show faculty email addresses or contact information through faculty pages, but their website allows them to send a message using a web form. For Assiut, Sohag, and Benha, most of their faculty members have web pages that provide academic and personal email, which explains the growth in the number of emails sent. About 2214 emails were returned for different causes, such as incorrect mailing addresses, bulk mail delays, and mail service non delivery. Six were left out of the research because of their unwillingness to participate in the survey. Descriptive statistics were computed using SPSS (version 22) to summarize the demographic data. Inferential statistics such as the independent and chi-square test were used to achieve the study aims. *P*-value ≤ 0.5 is the accepted significance level. An overwhelming majority (54.4%) of participants were female 210 and 176 were male (45.6%).

## Results

### Faculty members’ demographic characteristics

The demographic characteristics of the survey respondents were identified, as well as their implications, in this section. As Table 2 shows, most of the respondents (22.8%) are faculty members (36–40 age), and 18.7% of the respondents are younger (31–35 age), and (18.4%) are older faculty members (over 50).

**Table 2:** Demographic characteristics of respondents (*n* = 386)

Variable	Category	*n*	%
**Gender**	Male	176	45.6
	Female	210	54.4
	**Total**	**386**	**100**
**Age**	< 25	1	0.3
	26–30	37	9.5
	31–35	72	18.7
	36–40	88	22.8
	41–45	65	16.8
	46–50	52	13.5
	Over 50	71	18.4
	**Total**	**386**	**100**
**Academic job title**	Professor	80	20.7
	Assistant Professor	75	19.4
	Lecturer	119	30.8
	Assistant Lecturer	71	18.4
	Demonstrator	17	4.5
	Resident	15	3.9
	Visiting Resident	9	2.3
	**Total**	**386**	**100**
**Years of Experience**	< 3 years	34	8.8
	3–5 years	37	9.6
	6–10 years	67	17.4
	More than 10 years	248	64.2
	**Total**	**386**	**100**

Faculty members were asked to rate their job titles as seen in Table 2; most respondents classified themselves as lecturer (30.8%), and nearly 21% of the respondents classified themselves as professors. The rest of the respondents were distributed between the positions of assistant professor (19.4%) and assistant lecturer (18.4%), and the latter three groups (Demonstrator, Resident, Visiting Resident) represented low ratios.

We shall see that years of experience are essential in explaining some aspects of knowledge sharing behavior and faculty members’ attitudes. Table 2 shows that most respondents had more than ten years (64.2%) of experience.

Respondents were asked to list their academic fields of specialty; 6.7% of them were from Endemic Medicine, whereas 5.7% represented Internal Medicine. Participants who chose “Other” identified their specialty as follows: Hematology, Nutrition, Occupational and Environmental Medicine, Phoniatrics, Public Health and Preventive Medicine, Surgery, and Hepatology and Gastroenterology. Table 3 shows the specialty of the respondents.

**Table 3:** Participants’ responses to specialty (*n* = 386)

Specialty	*n*.	%	Specialty	*n*.	%
Anatomy &Embryology	16	4.1	Internal Medicine	22	5.7
Anesthesia and Surgical Intensive Care	13	3.4	Medical Physiology	10	2.6
Cardiology	10	2.6	Medical Biochemistry and Molecular Biology	6	1.6
Cardiothoracic Surgery	4	1.0	Medical Microbiology and Immunology	19	5.0
Chest Medicine	12	3.1	Medical Parasitology	9	2.3
Clinical and Chemical Pathology	16	4.1	Medical Pharmacology	8	2.1
Clinical Oncology and Nuclear Medicine	13	3.4	Neurology and Psychiatry	11	2.8
Community, Occupational and Environmental Medicine	16	4.1	Neurosurgery	5	1.3
Critical Care Medicine	3	0.8	Obstetrics and Gynecology	9	2.3
Dermatology, Venereology and Andrology	8	2.1	Oncology	3	0.8
Diagnostic and Interventional Radiology	5	1.3	Ophthalmology	13	3.4
Endemic Medicine	26	6.7	Orthopedic Surgery	13	3.4
Family Medicine	7	1.8	Pathology	16	4.1
Forensic Medicine and Clinical Toxicology	19	4.9	Pediatrics	12	3.1
General Surgery	13	3.4	Plastic Surgery	4	1.0
Geriatrics Medicine	1	0.3	Rheumatology and Rehabilitation	8	2.1
Histology	11	2.8	Other	25	6.5

About 74% of those surveyed indicated they were actively participating in medical practice during the COVID-19 pandemic, and (26.4%) were not.

### B. Knowledge sharing

**Fig. 1:**
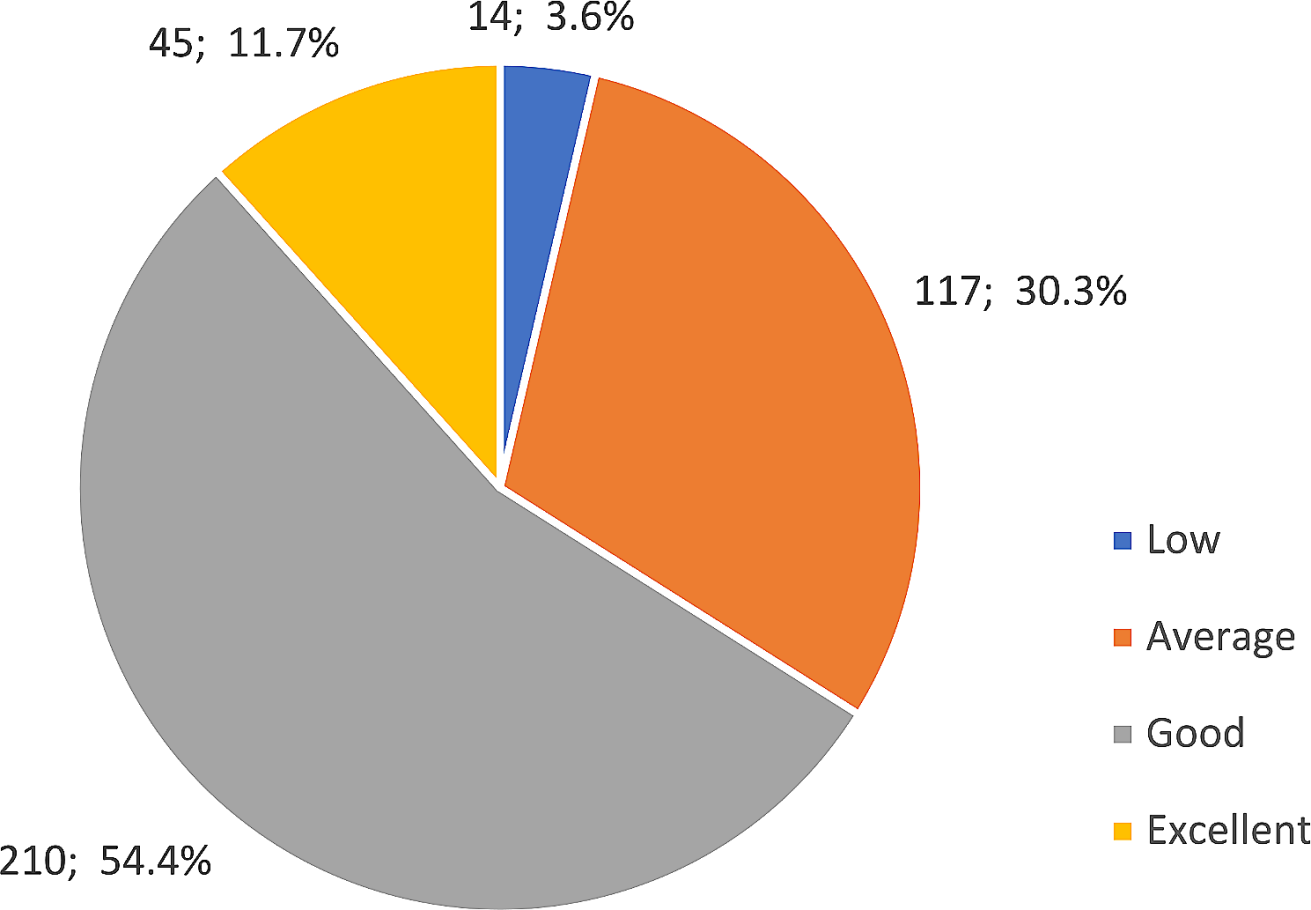
Participants’ responses to their knowledge of COVID-19 (*n* = 386)

More than half of the respondents (54.4%) indicated that their knowledge of COVID-19 was good. Figure 1 shows that only 11.7% of participants rated their knowledge excellent.

After over two years of mysteries that continue to tantalize healthcare workers, most participants rate their knowledge as good. Regarding gender, male participants have more knowledge about COVID-19 (*p*-value < 0.001) than females. (See Table 4)

**Table 4:** Relation between knowledge level and gender, age, academic job title

	Gender		Age							Academic job title						
Knowledge level of COVID-19	Male	Female	< 25	26–30	31–35	36–40	41–45	46–50	Over 50	Professor	Assistant Professor	Lecturer	Assistant Lecturer	Demonstrator	Resident	Visiting Resident
	%	%	%	%	%	%	%	%	%	%	%	%	%	%	%	%
Low	2.3	4.8	0	28.7	7.1	21.4	21.4	7.1	14.3	14.3	14.3	21.5	14.3	21.4	7.1	7.1
Average	23.3	36.2	0	15.4	22.2	29.1	10.3	12.8	10.2	12.8	19.7	26.5	25.6	7.7	4.3	3.4
Good	57.4	51.9	100	4.8	18.6	21.9	20.5	14.3	19.4	22.9	19	35.2	16.2	2.4	2.4	1.9
Excellent	17	7.1	0	11.1	13.3	11.1	15.6	13.3	35.6	33.3	22.2	24.4	11.2	0	8.9	0
	**Chi-square = 15.471 P-value = 0.001R**_**C**_ **= 0.196**		**Chi-square = 39.813 P-value = 0.002R**_**C**_ **= 0.306**							**Chi-square = 40.229 P-value = 0.002R**_**C**_ **= 0.307**						

* R_*C*_ *or Correlation Coefficient means the degree to which two variables have a linear relationship. Its value can range from − 1 to 1. A*

The study revealed a statistically significant relationship between age and respondents’ knowledge concerning COVID-19 (*P*-value = 0.002). It meant that as the age of faculty members increased, their knowledge also increased, as Table 4 shows.

The results in Table 4 showed that participants with a high academic degree, such as a professor, assistant professor, or lecturer, have more knowledge about COVID-19 than those with low level degrees (e.g., Assistant Lecturer, Demonstrator, Resident, and Visiting Resident).

Table 5 shows that male participants had higher knowledge than female participants because they were more actively involved in medical practices during the COVID-19 pandemic.

**Table 5:** Relation between participation in medical practice and gender

	Male		Female		Total	
	n	%	n	%	n	%
Yes	155	88.1	129	61.4	284	73.6
No	21	11.9	81	38.6	102	26.4
Total	176	100	210	100	386	100
Ch = 34.951		*P*-value = 0.000			Phi = 0.301	

**Fig. 2:**
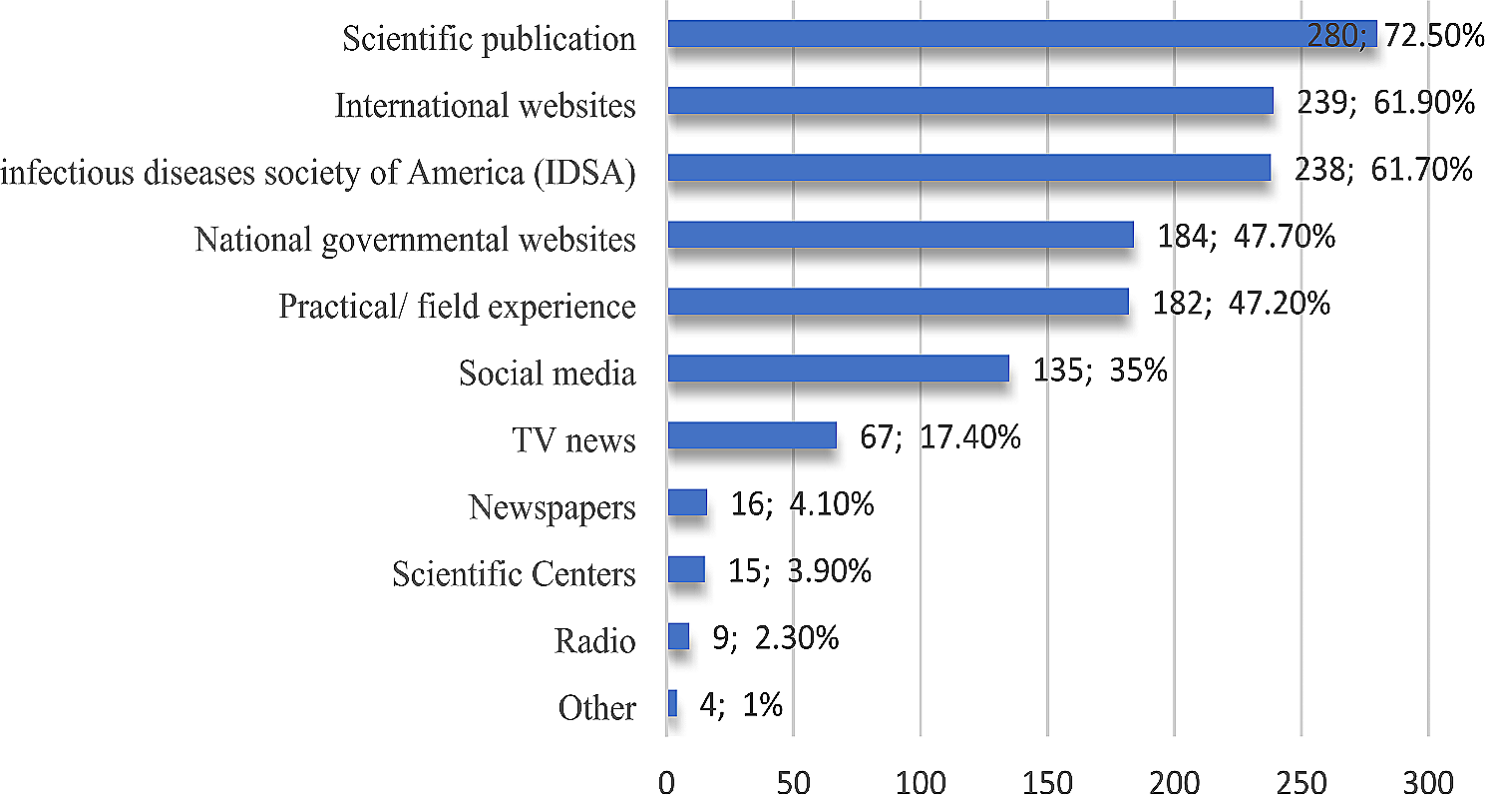
Participants’ responses to reliable sources of knowledge concerning COVID-19.

Figure 2 highlights that most of the participants (72.5%) reported that scientific publications (articles, conference papers, books, etc.), and (62%) international websites (i.e., WHO, Centers for Disease Control (CDC) were the most reliable source of their knowledge concerning COVID-19; however, reported other resources such as printed data and recommendations. Only four (1.5%) participants claimed that printed data and recommendations from the Egyptian Ministry of Health and Cardiology guidelines were the most reliable sources.

**Fig. 3:**
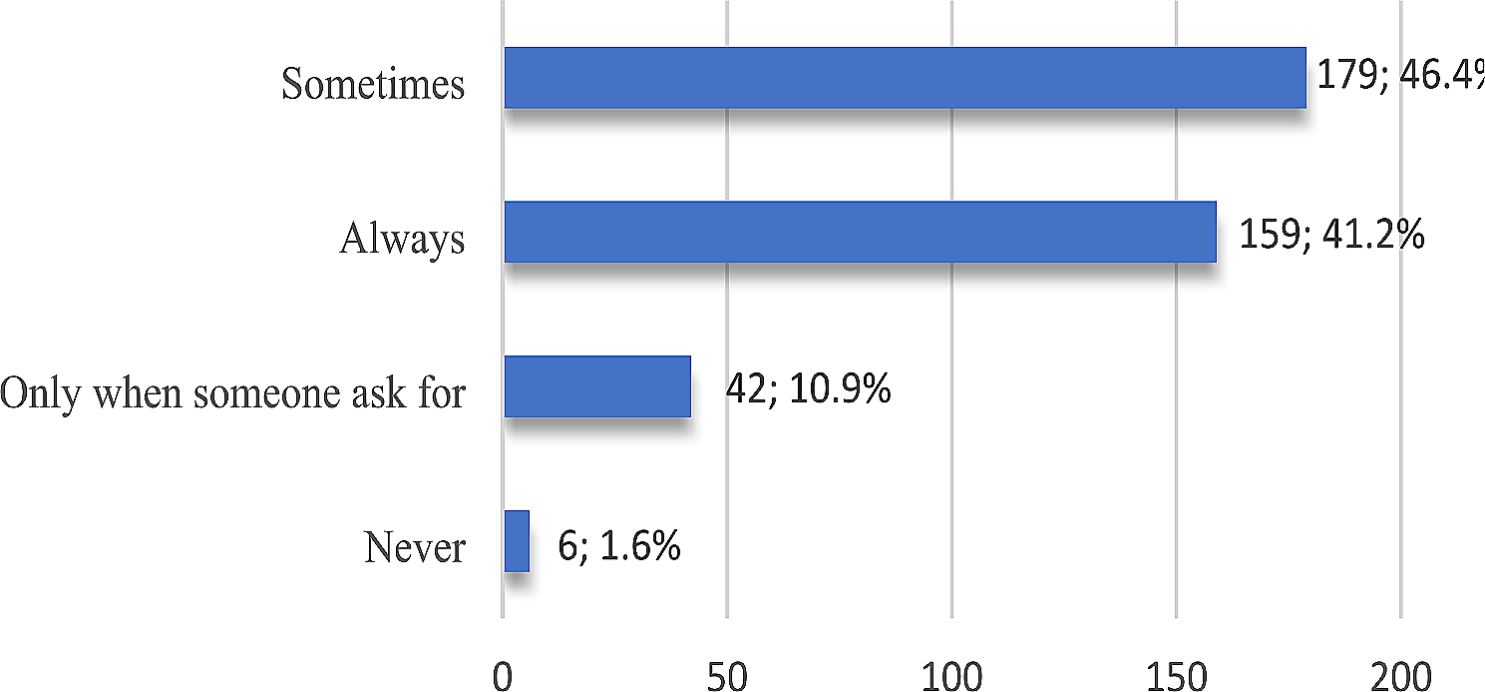
Participants’ responses to frequency of knowledge sharing (*n* = 386)

When participants were asked if they had heard of knowledge sharing, about 71% said yes, and 29% did not. Then, they were asked to record the frequency of their knowledge sharing behavior on a four-point Likert scale. As seen in Fig. 3, more than 46% (46.4%) stated that they sometimes share their knowledge, but only 1.6% stated that they never share (those who did not complete the rest of the questionnaire). They identified the reasons behind not sharing as follows: no one asked me to share my knowledge (4), unsure of its benefits (3), fear of misinformation (1), and uncertainty regarding novel diseases (1).

The analysis (Table 6) indicated that male and female participants were almost similar when answering the question “Do you share knowledge with your colleagues?” with no statistically significant difference.

**Table 6:** Relation between sharing knowledge with colleagues and gender, academic job title

	Gender		Academic job title						
Sharing knowledge with colleagues	Male	Female	Professor	Assistant Professor	Lecturer	Assistant Lecturer	Demonstrator	Resident	Visiting Resident
	%	%	%	%	%	%	%	%	%
Always	43.8	39	22	20.8	29.6	17.6	1.8	6.3	1.9
Sometimes	46.6	46.2	18.4	15.6	34.6	20.7	5	2.2	3.4
Only when someone ask for	8.5	12.9	26.2	28.6	19	11.9	11.9	2.4	0
Never	1.1	1.9	16.7	33.3	33.3	16.7	0	0	0
	**Chi-square = 2.534a**		**Chi-square = 23.684**						

Contrary to what could be expected, Table 6 showed that there is no statistically significant relationship between the academic appointment of participants and sharing knowledge with their colleagues. As a follow-up to the previous question, respondents who share knowledge (380 = 98.4%) were asked to address the factors that could affect sharing their knowledge. Table 7 indicates that 55% chose lack of time to share and 45% lack of organizational culture for knowledge sharing. Seven respondents who chose “other” indicated that lack of evidence-based knowledge, confidence in data, and workload were factors that affected them.

**Table 7:** Participants’ responses to factors that affect knowledge sharing

Factors	Frequency	Percent
Lack of time to share	209	55.0
Lack of organizational culture for knowledge sharing	171	45.0
Insufficient resources that can support opportunities for knowledge sharing	120	31.6
Lack of motivation and appreciation	114	30.0
Lack of awareness of the importance of knowledge sharing	91	23.9
Trust among staff	60	15.8
Unaware of recent communication technologies	40	10.5
Competition among colleagues	34	8.9
Gender and age differences	32	8.4
Other	7	1.9

*Note: multiple answers are permitted

Updating medical knowledge and becoming aware of the latest advancements are the most motivational factors underlying participants’ knowledge sharing during the COVID-19 pandemic. Nearly 71% chose to improve health services quality, while the rest of the respondents were distributed between (51.8%) to solve clinical issues, and (45.8%) to increase competence and learning.

As noted, 57.4% of participants specify the origin of the knowledge they share, both scholarly and non-scholarly. The remaining 36.8% are of scholarly origin, and only 5.8% are of non-scholarly origin.

### Explicit knowledge

**Fig. 4:**
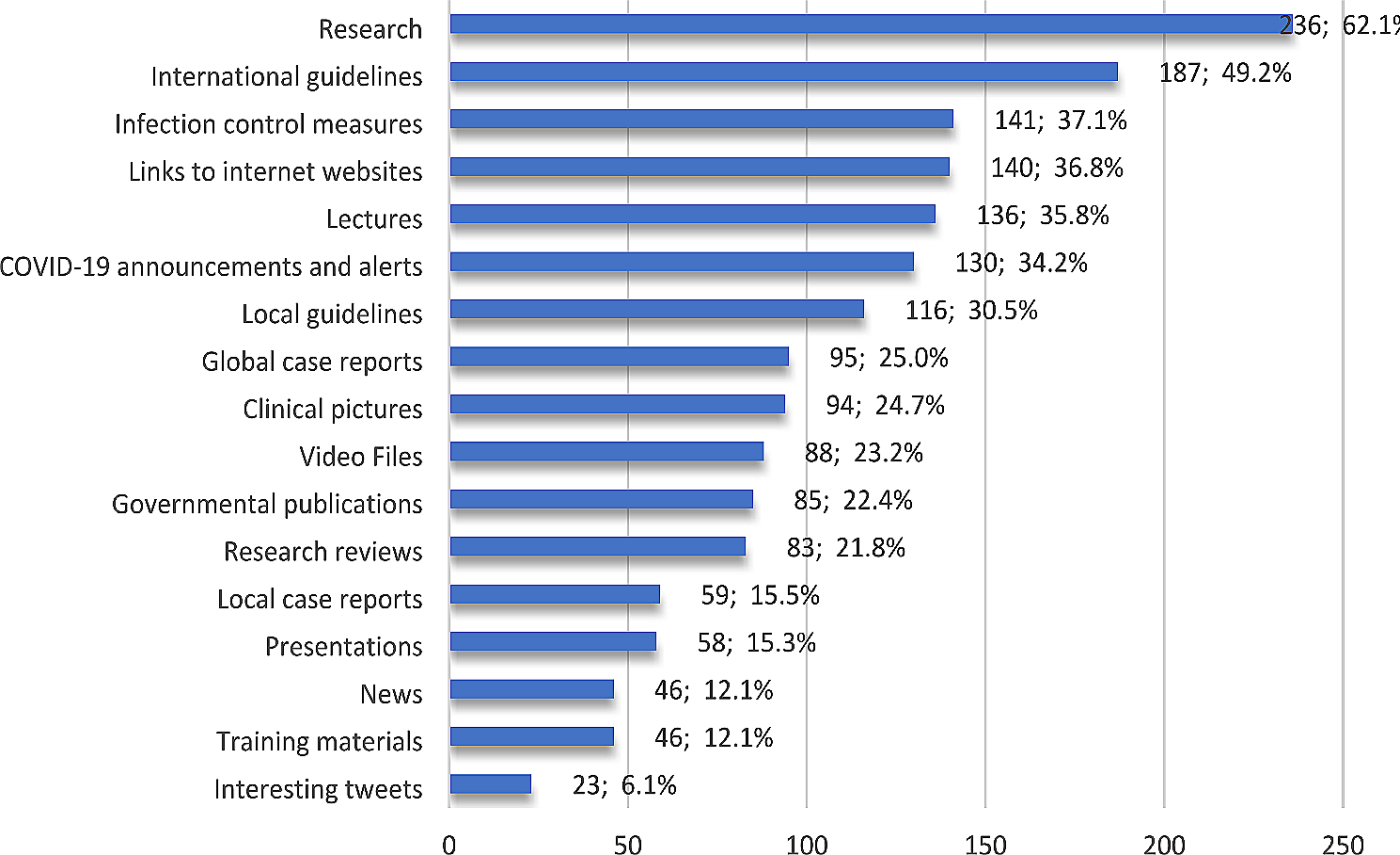
Participants’ responses to types of shared explicit knowledge

The current study identifies explicit knowledge as the formal and systematically stored, articulated, and disseminated information and published literature research such as journal papers, conference papers, books, etc. Figure 4 illustrates which type of COVID-19 explicit knowledge participants share. Research (62.1%) and international guidelines (49.2%) were the most explicit knowledge they shared. Contrary to this, the percentage of participants choosing interesting tweets was low (6.1%).

Interestingly, the results revealed a relationship between the type of COVID-19 explicit knowledge participants share and age (*p*-value < 0.001), as shown in Table 8.

**Table 8:** Relation between the type of shared explicit knowledge and age, years of experience

Type of explicit knowledge	Age							Years of experience			
	< 25	26–30	31–35	36–40	41–45	46–50	Over 50	Less than 3 years	3–5 years	5–10 years	More than 10 years
	%	%	%	%	%	%	%	%	%	%	%
Research (Journal papers, Conference papers, Books, …etc.)	12.5	15.7	13.4	16.1	15.2	16.6	15.8	10.8	15.1	15.9	15.7
Global case reports	12.5	3.7	5.2	5.6	6	3.9	5.4	4.2	5.5	4.8	5.4
Infection control measures	12.5	10.4	7.7	7.7	9.6	6.6	6.4	11.7	11	6.4	7.4
Lectures	12.5	9	7.7	8.2	6.6	9.6	5.6	6.7	7.5	9.9	7
Research reviews	0	3.7	3.6	4.3	6	4.4	5.4	5.8	4.8	3.5	4.7
Links to internet websites	12.5	4.5	6	8.2	7.9	10	8.6	8.3	4.8	4.8	8.8
International guidelines	0	10.4	10.4	11.7	10.6	9.2	9.7	12.5	10.3	9.6	10.4
COVID-19 announcements and alerts	12.5	5.2	6.3	7.9	7	7.4	8	5.8	6.8	6.4	7.6
Clinical pictures	0	5.2	8.2	4.3	3.6	3.1	5.9	5	5.5	7.3	4.7
Video Files	0	7.5	4.1	4.1	4.6	6.1	5.1	5.8	6.2	4.8	4.7
Governmental publications	0	2.2	4.9	4.8	4	7	4.6	1.7	4.1	6.1	4.7
Local guidelines	12.5	7.5	7.1	5.9	5.6	7.4	5.9	9.2	7.5	5.1	6.4
Local case reports	0	3.7	2.5	2.6	3.3	3.1	4.8	3.3	2.1	2.2	3.7
Training materials	0	2.2	4.1	2.8	1	2.2	2.4	0.8	2.1	5.1	2.1
Interesting tweets	0	2.2	2.2	1	1.3	0.4	0.8	2.5	1.4	1.6	1.1
Presentations	0	4.5	2.7	2.8	3.6	1.7	4.3	2.5	2.7	3.2	3.4
News	12.5	2.2	3.8	2	4	1.3	1.3	3.3	2.7	3.5	2.2
**Total**	**8**	**134**	**365**	**392**	**302**	**229**	**373**	**120**	**146**	**314**	**1223**
	**Chi-square = 146.322** **P-value = 0.001** R_C_**=0.524**					**Chi-square = 106.647** **P-value = 0.000** R_C_ =0.465					

* R_*C*_ *or Correlation Coefficient means the degree to which two variables have a linear relationship. Its value can range from − 1 to 1. A*

Table (8) shows a significant correlation between the type of COVID-19 explicit knowledge respondents share and years of experience (*p*-value = 0.000).

### Tacit Knowledge

**Fig. 5:**
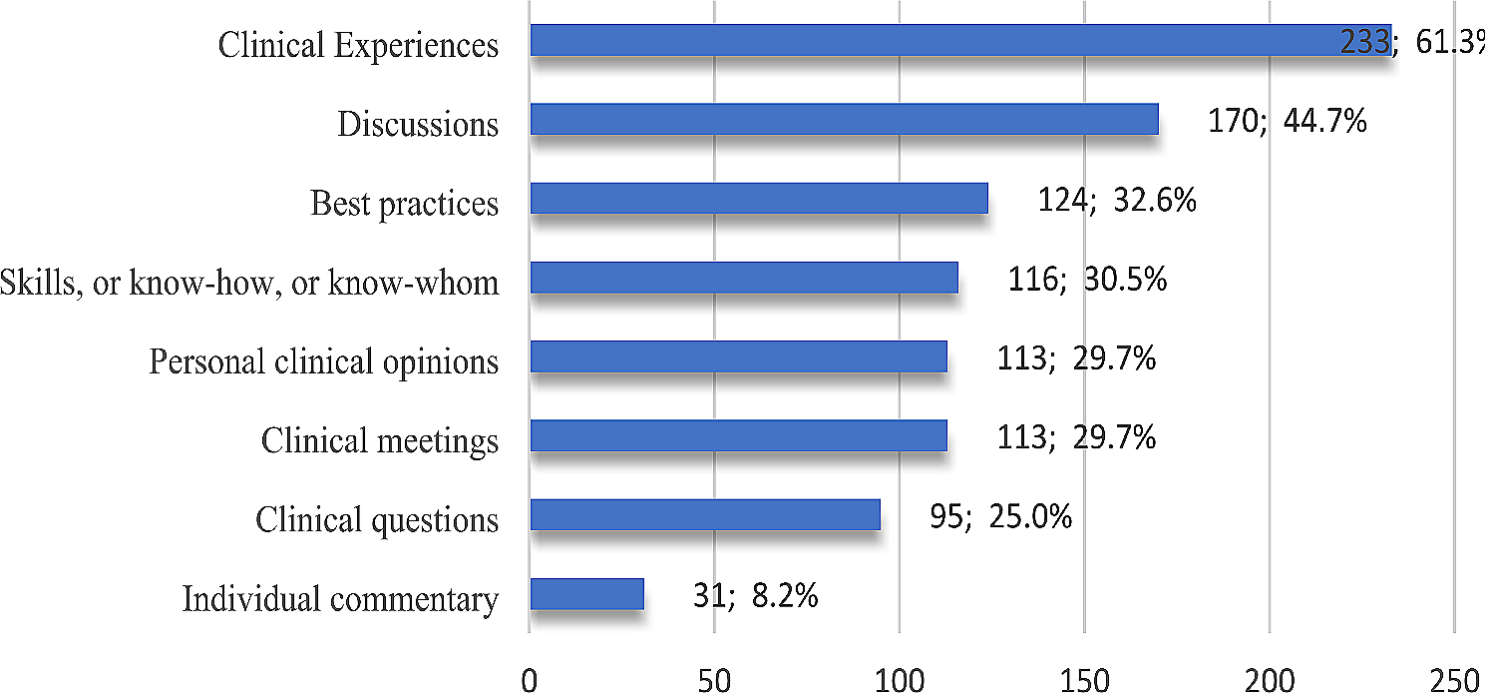
Participants’ responses to types of shared tacit knowledge

Regarding which type of COVID-19 tacit knowledge participants share (the knowledge embedded in people; they provide their knowledge and expertise and benefit from others), 61.3% chose to share their clinical experiences, and about 45% shared discussions. Figure 5 illustrates the breakdown by percentage of all respondents.

Table 9 shows a significant correlation between the type of COVID-19 tacit knowledge respondents share and academic appointment (*P*-value = 0.04).

**Table 9:** Relation between shared tacit knowledge type and academic job title

	Academic job title							
Tacit knowledge type	Professor	Assistant Professor	Lecturer	Assistant Lecturer	Demonstrator	Resident	Visiting Resident	Total
	%	%	%	%	%	%	%	
Best practices	11.8	13.3	12.3	13.3	6.3	8.7	26.1	125
Clinical meetings	14.2	10.6	11.7	7.7	9.4	13.0	17.4	113
Individual commentary	2.8	4.3	3.3	3.1	0	2.2	0	31
Clinical Experiences	19.3	21.8	26.3	26.7	25.0	19.6	13.0	233
Personal clinical opinions	15.6	10.1	10.7	9.7	9.4	15.2	0	113
Clinical questions	9.0	8.0	1 0	9.2	15.6	13.0	8.7	95
Discussions	17.0	18.1	17.0	17.4	15.6	17.4	8.7	170
Skills, or know-how, or know-whom	10.4	13.8	8.7	12.8	18.8	10.9	26.1	116
Total	**212**	**188**	**300**	**195**	**32**	**46**	**23**	**996**
Chi-square = 66.338	**P-value = 0.04**				R_**C**_**=0.382**			

* R_*C*_ *or Correlation Coefficient means the degree to which two variables have a linear relationship. Its value can range from − 1 to 1. A*

### Sort of shared knowledge

**Fig. 6:**
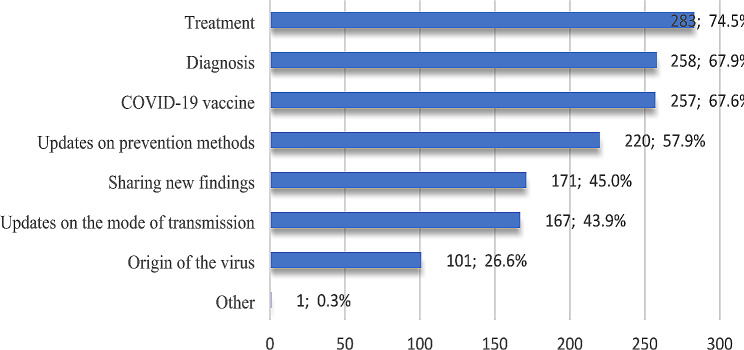
Participants’ responses to sort of shared tacit knowledge

Figure 6 below provides a further breakdown of the sort of COVID-19 knowledge faculty members shared; it is not surprising that about 75% of participants shared knowledge about treatment, COVID-19 diagnosis (67.9%), and vaccine (67.6%), which represented the most sorts of shared knowledge. One participant stated another type of knowledge, the “Mechanism of action”.

### Knowledge sharing mechanisms

**Fig. 7:**
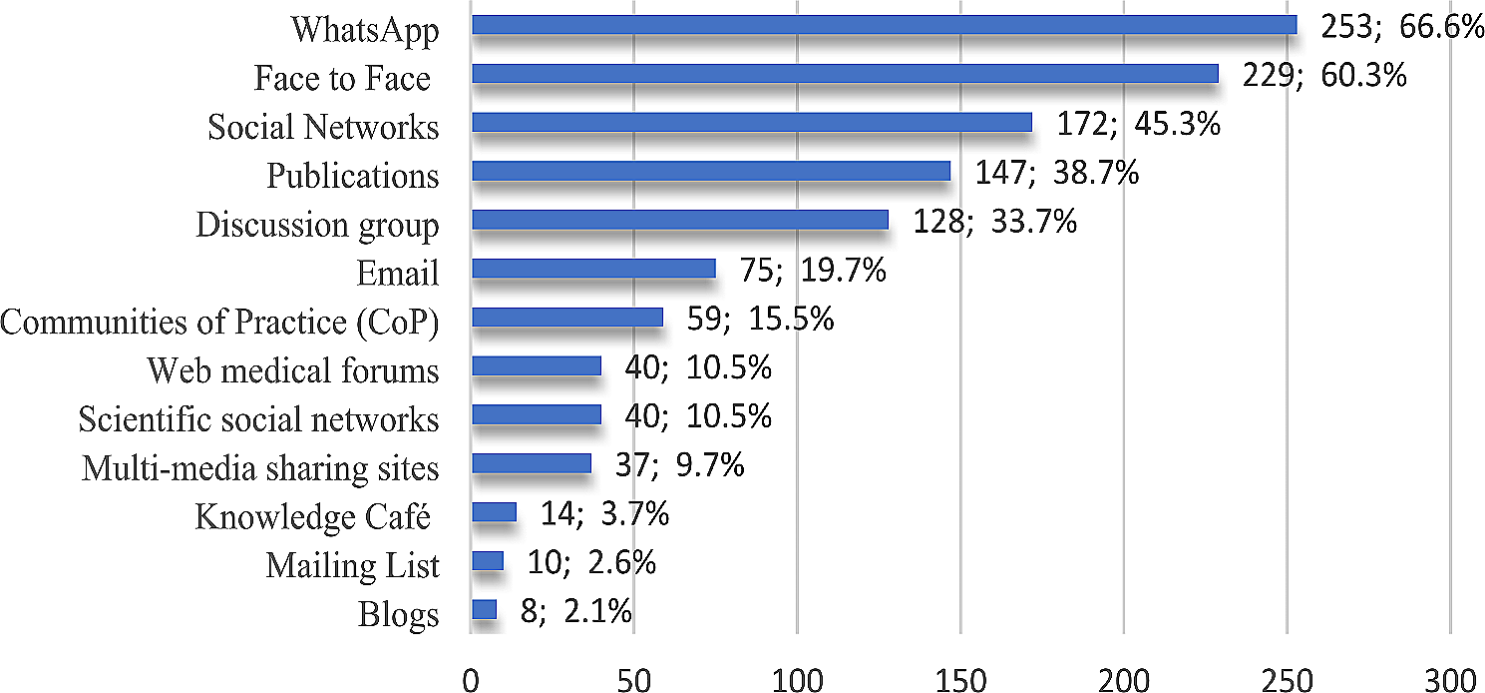
Participants’ responses to knowledge sharing Mechanisms

Each mechanism mentioned in Fig. 7 has its pros and cons. In general, 67% of participants in our survey reported that the WhatsApp messaging application was the most knowledge sharing channel they used.

Although wikis may offer a way to share knowledge and encourage faculty members to communicate instantaneously, contribute easily, and motivate collaborative work, none of the participants use wikis as a mechanism for sharing knowledge.

## Discussion

This study explores the knowledge-sharing behaviors of staff members in faculties of Medicine in Egyptian universities during the COVID-19 pandemic. It may have been expected that older faculty members with experience would be more likely to share their knowledge; on the contrary, younger and middle-aged represent most of the respondents who were more willing to share. Their intention to share knowledge may be influenced by the desire to raise the quality of their research and teaching and gain more organizational knowledge, and they may expect that by sharing their knowledge, other faculty members will reciprocate. Unsurprisingly, Endemic Medicine faculty members pay more attention to participating in the study. This aligns with findings from the Nature survey [26], which stated that almost 90% of scientists working on coronavirus think it will become endemic. When the pandemic first started, there was not much knowledge regarding COVID-19 and the virus, so only about 12% of participants rated their knowledge regarding COVID-19 as excellent. Interestingly, male participants have higher knowledge than females because males are more likely to practice appropriately most of the time [27].

The present study showed that as the age of faculty members increased, the amount of their knowledge also increased; this correlates with the findings of Taie [28], which revealed that physicians with work experience years have more excellent knowledge. In addition, the study revealed that scientific publications and international websites were the most reliable sources of knowledge concerning COVID-19 among participants. This correlates with the results of Abou-Abbas [15], which show that 85% of physicians in Lebanon used official international and governmental websites (such as WHO) as primary sources of information about COVID-19. Also, the results of a global survey mentioned that most physicians (51%) indicated official government websites as their most-used source during the COVID-19 pandemic and were the primary sources of knowledge [29].

Expectedly, scientific publications are reliable information sources that play an essential role in times of crisis. It is interesting to note that one-third of the participants did not hear of knowledge sharing. In some cases, participants who practice knowledge sharing do not know the exact meaning of what they do when exchanging their experiences and information with others. Numerous studies have been carried out to identify some of the obstacles impeding knowledge-sharing efforts among faculty members. Adamseged and Hong [30] stated that one of the biggest obstacles was people’s unwillingness to impart knowledge as they were unsure whether someone else needed knowledge or did not know how to proceed. Few faculty members in our study did not share knowledge because no one asked them to share, and they were unsure of its benefits, fear of misinformation, and uncertainty regarding novel diseases.

According to Ning, Fan, and Feng [31], professors and assistant professors have much knowledge and expertise and are more willing to share it with others. On the other hand, this group can have a knowledge-sharing response in which they seek to hoard knowledge because they believe that unique knowledge can be a source of strength. Contrary to what could be expected, our study showed that there is no statistically significant relation between the academic appointment of participants and sharing knowledge with their colleagues. The current study revealed that lack of time to share and lack of organizational culture for knowledge sharing were the most significant factors that could affect sharing their knowledge. Furthermore, 30% of participants noted that knowledge sharing is impacted by lack of motivation and appreciation. This might be because of workload, work stress, limited financial resources, inadequate infrastructure, as well as poor working environment. Similarly, a study by Moahi and Bwalya [22] showed that healthcare professionals in developing countries face many problems that affect their ability to effectively share knowledge. They are invariably overworked with large patient loads. Attending to patients leaves very little time for knowledge sharing. This is supported by the study by Bhatti [32], who claimed that knowledge sharing is very important in the healthcare industry, but the philosophy of intended performance restricts this knowledge sharing. The fundamental cause is the perception of threat in the individual’s mind of losing or giving away something belonging to him. Numerous studies have also demonstrated a variety of challenges that affect knowledge sharing practices. For instance, Asemahagn [33] found that knowledge and experience-sharing practices among Ethiopian health professionals are influenced by trust in others’ knowledge, motivation, supportive leadership, work satisfaction, awareness, willingness, and resource allocation. Assem [34] found that lack of trust, technological facilities, and organizational policy were some of the challenges healthcare professionals faced in sharing knowledge in the Ghanaian healthcare sector. Tan [35] stated that trust, organizational rewards, organizational culture, knowledge management system quality, openness in communication, and face-to-face interactive communication influence members’ knowledge sharing in Malaysian research universities. Ghodsian et al. [36] mentioned that professors’ attitudes toward knowledge transfer and sharing are the most relevant elements at the Tehran University of Medical Sciences. A study by Dessie [37] revealed that healthcare workers at public hospitals in North Shoa, Amhara, practiced knowledge-sharing poorly for several reasons, including access to information and communication technology infrastructure, familiarization with available technologies, and trust between staff and knowledge-sharing opportunities. Adeyelure, Kalema, and Motlanthe [38] reported that time constraints were a common challenge to all healthcare practitioners within the South African healthcare system.

Updating medical knowledge and becoming aware of the latest advancements are the motivational factors underlying participants’ knowledge sharing during the COVID-19 pandemic, which means that self-interest drives knowledge sharing among participants and the role of their academic institutions in motivation is not clear. As reported by Adamseged and Hong [30], the lack of reward/motivation/recognition for would-be knowledge sharers is one of the challenges an organization may create that hampers knowledge sharing because some individuals have an innate desire to share their knowledge, while others need external motivations. Accordingly, efforts to promote knowledge to everyone in need would be hampered if there were no available means through which higher education institutions could reward or recognize faculty members who actively participate in knowledge sharing.

Regarding the origin of shared knowledge by participants, few of them said that they share a non-scholarly origin of knowledge. That underlines the importance of scholarly knowledge during global health crises because of its quality in research and publishing. Research and international guidelines for COVID-19 were the most type of explicit knowledge participants shared. Contrary to this, a low percentage of participants chose interesting tweets, indicating that despite the considerable amount of reliable information on social media generated during the COVID-19 outbreak, few faculties are interested in writing tweets to share their knowledge. However, social media is used by higher education institutions to encourage student participation in class activities and information exchange as it makes knowledge sharing in the virtual world easy and constant [39].

A strong correlation was observed between age and type of COVID-19 explicit knowledge shared. Older faculty members preferred to share reliable resources like journal papers, conference papers, books, and international guidelines. On the other hand, younger participants preferred global case reports, COVID-19 announcements and alerts, local guidelines, and news. Regarding the latter, Gallotti et al. [40] asserted that as the public health crisis of COVID-19 spread and became an international concern, Twitter (as well as Facebook and Google) took action against the spread of unreliable/misleading news to favor reliable sources over unreliable sources. In addition, our findings show a correlation between the type of COVID-19 explicit shared knowledge and years of experience. Respondents who had a good level of experience of more than ten years had a specific type of knowledge to share, like research publications and international guidelines.

In the context of the current study, it was found that participants chose clinical experiences and discussions as the tacit knowledge they shared the most. This result was expected because medical education depends heavily on bedside teaching and discussion of patients’ cases. Despite the significance of “individual commentary,” which sums up or assesses what the doctor is observing, sensing, or hearing while doing the patient’s physical examination [41], only 8% stated that they share this type of tacit knowledge. Some kinds of tacit knowledge were more shared according to job titles, such as clinical experiences and discussions, and were more shared among higher job titles. At the same time, lower job titles shared best practices concerning COVID-19. It is worth noting that a set of best practices for the COVID-19 pandemic has been released on national and international levels, illustrating methods and techniques that increase efficiency and develop structured processes during the pandemic. It is the most practical and successful course of action for particular circumstance.

The study demonstrated that the majority of participants shared knowledge about treatment because, till the time of conducting this survey, there was no proven treatment against the virus. Then, the COVID-19 diagnosis and vaccine represented the most shared knowledge. Regarding the latter, many concerns among healthcare workers (HCWs) are raised regarding the benefits of vaccination, its effectiveness, side effects, and the differences between types of vaccines [42]. Concerning knowledge-sharing channels, more than two-thirds of participants identified WhatsApp messaging applications as the most common channel they used. This goes in line with the study of Attalla et al. [43], which indicated that WhatsApp is a fast alternative way of passing information and acquiring work-related knowledge among staff at the medical school at Management and Science University (MSU), Malaysia, compared to other channels like email. It is worth noting that WhatsApp is one of the most popular social media platforms in Egypt; as of January 2024, (72%) of internet users in the country used the platform [44]. This may be due to the advantages that WhatsApp has, like the friendly user interface, ease of use, different modes of communication, comfortable sharing of sensitive information, greater diversity of participants, and the ability to send private and encrypted messages between individuals [45].

Not all knowledge-sharing mechanisms are IT-based; face-to-face was the second most favorable channel for sharing knowledge, as most participants demonstrated. This is supported by the study by Diriba, Jimma, and Roba [46], in which they revealed that participants preferred to share knowledge through face-to-face interaction, either in a formal event such as a structured meeting or through an informal activity such as an opportunistic discussion. Similarly, Adeyelure, Kalema, and Motlanthe [38] explored that traditional face-to-face knowledge sharing is the most often used method to share knowledge. Social networks were stated as the third channel used by participants for sharing (60.3%). A similar pattern of results was obtained in various studies, indicating that social media is the preferred platform for sharing knowledge about the pandemic. A study by Abdel Wahed et al. [47] reported that social media is the most reliable source of information regarding COVID-19 among HCWs. Similarly, a study by Hussein et al. [48] stated that Egyptian physicians are active social media users for disseminating information during pandemics. Also, a study by Ali and Hamed [49] reported that a group of Egyptian physicians had good knowledge about the SARS-CoV-2 pandemic, which was mainly gained through social media channels, despite the importance of the community of practice in collecting and sharing knowledge, as in “C-WorKS” in England, which developed in 2020 during COVID-19, only about 16% of participants chose community of practice as their channel for sharing knowledge.

## Conclusion

Higher education institutions are involved in producing knowledge and sharing knowledge is crucial to this process. However, many have not accepted the necessity of knowledge sharing among their faculty members as a necessary attempt to successfully impart knowledge to students and society [30].

To summarize, the findings of this study show more positive attitudes toward knowledge sharing among faculty members. On the other hand, about one-third of the participants have not heard of knowledge sharing, which means that awareness regarding knowledge sharing needs to be created among faculty members in medical education institutions. Respondents indicated that scientific publications and international websites were the most reliable source of their knowledge concerning COVID-19.

Notably, many medications are being tested, and only two have been approved by the FDA [50]. Many vaccines are available, and there are no specific clinical features for the virus; respondents’ knowledge about treatment, diagnosis, and vaccines attracts attention when they share knowledge with their colleagues. The findings of this study suggested there was a need for more efforts from the Egyptian Ministry of Health in collaboration with medical schools and research centers toward building a national platform for sharing scientific research results and reliable content related to COVID-19 among researchers.

The current study reports that lack of time is one of the top challenges affecting knowledge-sharing behavior among faculty members in the faculties of Medicine. Time is a fundamental issue for universities to tackle the crisis properly and find out how to deal with the situation as educational institutions and scientific research bodies. According to the study results, the faculties of Medicine in Egypt need to improve their organizational culture to enhance the knowledge sharing behavior of their staff and develop a knowledge sharing strategy for better quality medical education. Finally, the study discussed some effective mechanisms of knowledge sharing among faculty members during the COVID-19 pandemic encompassing WhatsApp, face-to-face, and social networks.

A significant limitation of this study is the distribution of the email survey questionnaire, which represents a low response rate. In addition, faculty members in Medicine always have no time to be involved in such surveys. Besides, the study revealed participants’ self-assessment of their knowledge about COVID-19, by asking them only one question to reflect their level of knowledge. The disadvantage of this approach is that participants may not be able to accurately assess their knowledge. Future research agendas recommend exploring knowledge management in the Egyptian faculties of Medicine, knowledge sharing among faculty staff in private Medical schools in Egypt, evidence-based knowledge in Egyptian Medical education, and the impact of the Infodemic on scholarly communication during health crisis.

### Electronic supplementary material

Below is the link to the electronic supplementary material.

Supplementary Material 1

## Data Availability

The datasets generated and analyzed during the current study are not publicly available because they contain information about staff members in medical schools that they did not consent to share publicly at the individual level. Still, aspects of the data set may be available from the corresponding author upon reasonable request.

## Abbreviations

COVID-19: Severe acute respiratory syndrome coronavirus (SARS-CoV-2)
CDC: Centers for Disease Control and Prevention
FDA: U.S. Food and Drug Administration
HCWs: Healthcare Workers
WHO: World Health Organization
