# Supplementary material for: Knowledge sharing among academics from Egyptian medical schools during the COVID-19 pandemic
Source: BMC Med Educ. 2024 Jun 1;24:608. doi: 10.1186/s12909-024-05502-2 (PMC11143613; doi:10.1186/s12909-024-05502-2)
Supplement: Supplementary file 1 — Supplementary Material 1 [file 12909_2024_5502_MOESM1_ESM.pdf]

Annex: Questionnaire: Knowledge Sharing among Academics from Egyptian Medical schools during the COVID-19 Pandemic

Dear Colleague,

Please read the information about the study below and indicate whether you would like to participate or not. This questionnaire is being conducted as part of a study which is investigating knowledge sharing among Academics from Egyptian Medical schools during the COVID-19 Pandemic. knowledge sharing means the process of transferring knowledge locked in the human mind from one person to another on the same topic.

The research is being carried out by Healthcare Informatics Research Group (HIRG) at Helwan University. All the answers you provide to the following questions will be treated confidentially and held securely. This questionnaire is completely anonymous and will not collect individually identifiable information. The questionnaire should take around (5-10) minutes to complete. Your agreement to participate grants us the use of your anonymized data for dissemination purposes.

This questionnaire has been approved by the Research Ethics Committee for Human and Animal Research at the faculty of Medicine, Helwan University (No. 17-2021).

If you have questions regarding this study, you may contact Prof. **Amany Mohamed Elsayed** by email: amany03@gmail.com

Do you agree to participate in this study? If your answer is **"Yes,** you can go to the questionnaire, and if **"NO"** questionnaire will not begin.

☐ Yes

☐ No

Part (1): Demographic Information

– **Name (optional):**

– **Gender:**

- ☐ Male
- ☐ Female

– **Age:**

- ☐ 25 and under
- ☐ 26-30
- ☐ 31-35
- ☐ 36-40
- ☐ 41-45
- ☐ 46-50
- ☐ Over 50

– **Academic job title:**

- ☐ Professor
- ☐ Assistant Professor
- ☐ Lecturer
- ☐ Assistant Lecturer
- ☐ Demonstrator
- ☐ Resident
- ☐ Visiting Resident

– **Department/Specialty:**

- ☐ Anatomy &Embryology
- ☐ Anesthesia and Surgical Intensive Care
- ☐ Cardiology
- ☐ Cardiothoracic Surgery
- ☐ Chest Medicine
- ☐ Clinical and Chemical Pathology
- ☐ Clinical Oncology and Nuclear Medicine
- ☐ Community, Occupational and Environmental Medicine
- ☐ Critical Care Medicine
- ☐ Dermatology, Venereology and Andrology
- ☐ Diagnostic and Interventional Radiology
- ☐ Endemic Medicine
- ☐ Family Medicine
- ☐ Forensic Medicine and Clinical Toxicology
- ☐ General Surgery
- ☐ Geriatrics Medicine
- ☐ Histology
- ☐ Internal Medicine
- ☐ Medical Physiology
- ☐ Medical Biochemistry and Molecular Biology
- ☐ Medical Microbiology and Immunology
- ☐ Medical Parasitology

- ☐ Medical Pharmacology
- ☐ Neurology and Psychiatry
- ☐ Neurosurgery
- ☐ Obstetrics and Gynecology
- ☐ Ophthalmology
- ☐ Orthopedic Surgery
- ☐ Otorhinolaryngology
- ☐ Pathology
- ☐ Pediatric Surgery
- ☐ Pediatrics
- ☐ Plastic Surgery
- ☐ Rheumatology and Rehabilitation
- ☐ Other, please specify:

– **Years of Experience:**

- ☐ Less than 3 years
- ☐ 3-5 years
- ☐ 6-10 years
- ☐ More than 10 years

Part (2): knowledge sharing practices.

Q1. Are you actively participating in medical practice during COVID-19 pandemic?

- ☐ Yes
- ☐ No

Q2. How you describe your knowledge concerning COVID-19?

- ☐ Low
- ☐ Average
- ☐ Good
- ☐ Excellent

Q3. Please choose the most reliable sources of your knowledge concerning COVID-19 below. (You can choose up to 4 sources only)

- ☐ Scientific publication (Article, Conference paper, book, etc.)
- ☐ Practical/ field experience
- ☐ National governmental websites (i.e., The Ministry of Health, Scientific Centers, etc.)
- ☐ International websites such as: WHO, Centers for Disease Control (CDC) , infectious diseases society of America (IDSA)…etc.
- ☐ Social media (Facebook, Twitter, Instagram, YouTube…etc.)
- ☐ TV news
- ☐ Radio
- ☐ Newspapers

☐ Other, please specify:

Q4. Do you hear of knowledge sharing?

☐ Yes

☐ No

Q5. Do you share knowledge with your colleagues?

☐ Always

☐ Sometimes

☐ only when someone asks for

☐ Never

Q6. If the answer is "Never" Please, explain why? (You can choose more than one answer)

☐ Unwillingness to share experiences

☐ Fear of loss of personal competitiveness

☐ Fear of misinformation

☐ No one ask me to share my knowledge

☐ Uncertainty regarding novel diseases

☐ Top Management does not support knowledge sharing

☐ Unsure of its benefits

☐ Other, please specify:

Q7. Which factors could affect sharing your knowledge? (You can choose more than one answer)

☐ Lack of time to share

☐ Competition among colleagues

☐ Lack of organization culture for knowledge sharing

☐ Trust among staff

☐ Lack of awareness of the importance of knowledge sharing

☐ Unaware of recent communication technologies

☐ Lack of motivation and appreciation

☐ Gender and age differences

☐ Insufficient of resources that can support opportunities for knowledge sharing

☐ Other, please specify:

Q8. What are the motivations that underlie your knowledge-sharing during COVID-19 pandemic? (You can choose more than one answer)

☐ To improve health services quality.

☐ Updating medical knowledge and getting aware of the latest advancements

☐ Solving clinical issues

☐ Increase competence and learning

☐ Other, please specify:

Q9. Please specify the origin of knowledge you are sharing.

☐ Scholarly origin

☐ Non scholarly origin

☐ Both

Q10. Which type of COVID-19 explicit knowledge do you share? (You can choose more than one answer)

Explicit knowledge: (formal and systematically stored, articulated, and disseminated information and published literature)

☐ Research (Journal papers, Conference papers, Books, …etc.)

☐ Lectures

☐ Video Files

☐ Training materials

☐ Links to internet websites

☐ Global case reports

☐ Local case reports

☐ Infection control measures

☐ COVID-19 announcements and alerts

☐ Clinical pictures

☐ Governmental publications

☐ Local guidelines

☐ International guidelines

☐ Interesting tweets

☐ Presentations

☐ Research reviews

☐ News

☐ Other, please specify:

Q11. Which type of COVID-19 tacit knowledge do you share? (You can choose more than one answer)

Tacit knowledge: (knowledge embedded in people, they provide their knowledge and expertise and get benefit from knowledge of others).

☐ Clinical Experiences

☐ Skills, or know-how, or know-whom.

☐ Personal clinical opinions

☐ Clinical meetings

☐ Best practices

☐ Discussions

☐ Clinical questions

☐ Individual commentary

☐ Other, please specify:

Q12. What sort of COVID-19 knowledge did you share? (You can choose more than one answer)

- ☐ Origin of the virus
- ☐ Diagnosis
- ☐ Treatment
- ☐ Sharing new findings
- ☐ Updates on the mode of transmission
- ☐ Updates on prevention methods
- ☐ COVID-19 vaccine
- ☐ Other, please specify:

- ☐ **Q13. Through which of the following means did you share knowledge? (Knowledge sharing platforms)** (You can choose more than one answer)
- ☐ Publications (Journals, Books, conferences, …etc.)
- ☐ Face to Face
- ☐ Social Networks (Facebook, twitter, LinkedIn, ... etc.)
- ☐ WhatsApp
- ☐ Email
- ☐ Mailing List
- ☐ Discussion Group
- ☐ Web medical forums
- ☐ Blogs
- ☐ Multi-media sharing sites (YouTube, Vimeo, iTunes, other)
- ☐ Wiki (a website that allows users to add and update content collaboratively such as Wikipedia)
- ☐ Scientific social networks (ResearchGate, Academia, ...)
- ☐ Communities of practice (group of people sharing a common interest and working together for a period to explore ways of working in a specific area of knowledge).
- ☐ Knowledge Café (a group of people who meet to collect knowledge, learn from each other, sharing ideas on a topic)
- ☐ Other, please specify:
